# P-1250. Comparison of Eight Machine Learning-based Covariate Risk Prediction Models for Vancomycin-associated Acute Kidney Injury during Initial Dosing

**DOI:** 10.1093/ofid/ofaf695.1441

**Published:** 2026-01-11

**Authors:** Moeko Iida, Yasuhiro Horita, Masato Noda, Minami Asaoka, Yoshinori Hisada, Masaya nagamizu, Kaori Tsuzuki, Masaharu Kudo, Sakurako Muramatsu, Yuki Nomura, Chiharu Wachino, Masami Kawahara, Nobuyuki Morishita, Masahiro Kondo, Yuji Hotta, Atsushi Nakamura, Yoko Furukawa-Hibi

**Affiliations:** Graduate School of Medical Sciences, Nagoya City University, Nagoya, Aichi, Japan; Graduate School of Medical Sciences, Nagoya City University, Nagoya, Aichi, Japan; Nagoya City University East Medical Center, Nagoya, Aichi, Japan; Graduate School of Medical Sciences, Nagoya City University, Nagoya, Aichi, Japan; Nagoya City University West Medical Center, Nagoya, Aichi, Japan; Nagoya City University West Medical Center, Nagoya, Aichi, Japan; Nagoya City University East Medical Center, Nagoya, Aichi, Japan; Nagoya City University Midori Municipal Hospital, Nagoya, Aichi, Japan; Aichi Gakuin University, Nagoya, Aichi, Japan; Nagoya City University Hospital, Nagoya, Aichi, Japan; Nagoya City University East Medical Center, Nagoya, Aichi, Japan; Aichi Gakuin University, Nagoya, Aichi, Japan; Nagoya City University West Medical Center, Nagoya, Aichi, Japan; Nagoya City University East Medical Center, Nagoya, Aichi, Japan; Graduate School of Medical Sciences, Nagoya City University, Nagoya, Aichi, Japan; Nagoya City University Hospital, Nagoya, Aichi, Japan; Graduate School of Medical Sciences, Nagoya City University, Nagoya, Aichi, Japan

## Abstract

**Background:**

Vancomycin is a key antibiotic for methicillin-resistant *Staphylococcus aureus* infections, and area under the concentration–time curve (AUC)-guided vancomycin dosing is recommended for reducing the risk of acute kidney injury (AKI). Recently, machine learning (ML) has proven useful and the techniques have rapidly progressed in the clinical pharmacology field. Several reports related to ML models for predicting vancomycin-associated AKI have been published; however, a ML model for predicting vancomycin-associated AKI based on AUC at an early stage of drug administration has not yet been devised. Therefore, we constructed a risk prediction model incorporating AUC and relevant risk factors such as concomitant drugs and comorbidities.

**Methods:**

We conducted a multicenter, retrospective, cohort study among hospitalized patients treated with vancomycin at three hospitals between April 1, 2019 and March 31, 2024. Vancomycin-associated AKI was defined based on the modified Kidney Disease Improving Global Outcomes criteria. Non-steady-state AUCs (AUC_0–24h_ and AUC_24–48h_), were calculated according to the actual dosing schedule using the TDM software program SAKURA-TDM (Horita Y *et al.*, TDM, 2023). Based on previous studies, 23 variables were included in the first step of model development. Risk prediction models were built using eight ML algorithms, and model performances were evaluated using accuracy, precision, recall, and F1 scores.

**Results:**

Five important features were identified: AUC_24–48h_, trough concentrations, and concomitant use of piperacillin/tazobactam, diuretics, or vasopressors. Among the evaluated ML models, Naïve Bayes and random forest exhibited equally high predictive performance with accuracy of 97.3% (95% confidence interval 93.1%–99.3%), precision of 83.3%, recall of 62.5%, and F1 score of 71.4%. The F1 scores in models without either AUC_24–48h_ or trough levels were lower than those in the original model (AUC_24–48h_ plus trough levels).

**Conclusion:**

We constructed a ML-based risk prediction model for vancomycin-associated AKI at an early stage of administration. Our results underscore the importance of incorporating both AUC_24–48h_ and trough levels in the development of such risk prediction models.

**Disclosures:**

All Authors: No reported disclosures

